# A comparison of conditional random fields and structured support vector machines for chemical entity recognition in biomedical literature

**DOI:** 10.1186/1758-2946-7-S1-S8

**Published:** 2015-01-19

**Authors:** Buzhou Tang, Yudong Feng, Xiaolong Wang, Yonghui Wu, Yaoyun Zhang, Min Jiang, Jingqi Wang, Hua Xu

**Affiliations:** 1 Department of Computer Science, Harbin Institute of Technology Shenzhen Guraduate, Shenzhen, Guangdong, China; 2 School of Biomedical Informatics, The University of Texas Health Science Center at Houston, Houston, Texas, USA; 3 Department of Pharmacy, the First Affiliated Hospital, Harbin Medical University Harbin, Heilongjiang, China

## Abstract

**Background:**

Chemical compounds and drugs (together called chemical entities) embedded in scientific articles are crucial for many information extraction tasks in the biomedical domain. However, only a very limited number of chemical entity recognition systems are publically available, probably due to the lack of large manually annotated corpora. To accelerate the development of chemical entity recognition systems, the Spanish National Cancer Research Center (CNIO) and The University of Navarra organized a challenge on Chemical and Drug Named Entity Recognition (CHEMDNER). The CHEMDNER challenge contains two individual subtasks: 1) Chemical Entity Mention recognition (CEM); and 2) Chemical Document Indexing (CDI). Our study proposes machine learning-based systems for the CEM task.

**Methods:**

The 2013 CHEMDNER challenge organizers provided a manually annotated 10,000 UTF8-encoded PubMed abstracts according to a predefined annotation guideline: a training set of 3,500 abstracts, a development set of 3,500 abstracts and a test set of 3,000 abstracts. We developed machine learning-based systems, based on conditional random fields (CRF) and structured support vector machines (SSVM) respectively, for the CEM task for this data set. The effects of three types of word representation (WR) features, generated by Brown clustering, random indexing and skip-gram, on both two machine learning-based systems were also investigated. The performance of our system was evaluated on the test set using scripts provided by the CHEMDNER challenge organizers. Primary evaluation measures were micro Precision, Recall, and F-measure.

**Results:**

Our best system was among the top ranked systems with an official micro F-measure of 85.05%. Fixing a bug caused by inconsistent features marginally improved the performance (micro F-measure of 85.20%) of the system.

**Conclusions:**

The SSVM-based CEM systems outperformed the CRF-based CEM systems when using the same features. Each type of the WR feature was beneficial to the CEM task. Both the CRF-based and SSVM-based systems using the all three types of WR features showed better performance than the systems using only one type of the WR feature.

## Background

Chemical compounds and drugs (together called chemical entities) embedded in scientific articles are crucial for many information extraction tasks in the biomedical domain, such as detection of drug-protein interactions and adverse drug reactions [1]. Recognizing chemical entities from biomedical literature is a typical named entity recognition (NER) task. Compared with other NER tasks, such as NER in newswire domain [2], biomedical NER in biomedical domain [3,4] and clinical NER in clinical domain [5], there are many unique challenges in chemical entity recognition. For example, a chemical entity may contain a number of long phrases and symbols. There are also a large number of hybrid entities that are partial, systematic and trivial.

A number of comprehensive chemical databases, such as PubChem [6], ChEBI [7] Jochem [8], ChemSpider [9], MeSH [10] and DrugBank [11] have been developed for various purposes, and could potentially be used as lexicons for chemical entity recognition. However, only a very limited number of chemical entity recognition systems have been developed and made publically available, probably due to the lack of large manually annotated corpora. The representative systems are Whatizit [12], OSCAR3/4 [13,14] and ChemSpot [15]. Whatizit uses dictionary lookup to recognize chemical entities. OSCAR3/4 are machine learning-based systems that utilize maximum entropy models [16] on the OSCAR corpus. ChemSpot is a hybrid system that combines a machine learning-based classifier on SCAI corpus [17,18] with a dictionary. Conditional random fields (CRF) [19] are used as the classifier in ChemSpot. So far, no comparative evaluation for different chemical entity recognition systems has been investigated on a standard corpus.

To accelerate the development of chemical entity recognition systems, The Spanish National Cancer Research Center (CNIO) and University of Navarra organized a challenge on Chemical and Drug Named Entity Recognition (CHEMDNER), as a part of BioCreative IV challenge (Track 2) [20-22]. The CHEMDNER challenge includes two individual subtasks: 1) Chemical Entity Mention recognition (CEM); and 2) Chemical Document Indexing (CDI). The subtask 1 is a typical named entity recognition task. The subtask 2 requires participants to rank chemical entities according to their importance in a chemical document. The challenge organizers provided manually annotated abstracts from PubMed (10,000 abstracts), of which 3,500 abstracts were used as a training set, 3,500 abstracts were used as a development set, and 3,000 abstracts were used as a test set.

In this paper, we described our systems for the CEM task. The systems first used a rule-based module for sentence boundary detection and tokenization, and then built machine learning classifiers based on CRF [19] and structured support vector machines (SSVM) [23] respectively. Both CRF and SSVM are state-of-the-art machine learning methods for NER, but SSVM has not been applied to chemical entity recognition yet. CRF is a discriminative undirected probabilistic graphical model, while SSVM is a discriminative model based on large margin theory. We also investigated the effects of three types of word representation (WR) features, generated by Brown clustering [24], random indexing [25] and skip-gram [26], on both the CRF-based and SSVM-based systems. WR is a new feature extraction technique that uses unsupervised learning algorithms to generate word-level features from an unlabeled corpus. Those WR features usually contain latent syntactic/semantic information of a word.

Our system was among the top ranked systems with an official micro F-measure of 85.05%. After fixing a bug caused by inconsistent features, the performance was marginally improved with a micro F-measure of 85.20%.

## Methods

**Figure 1:**
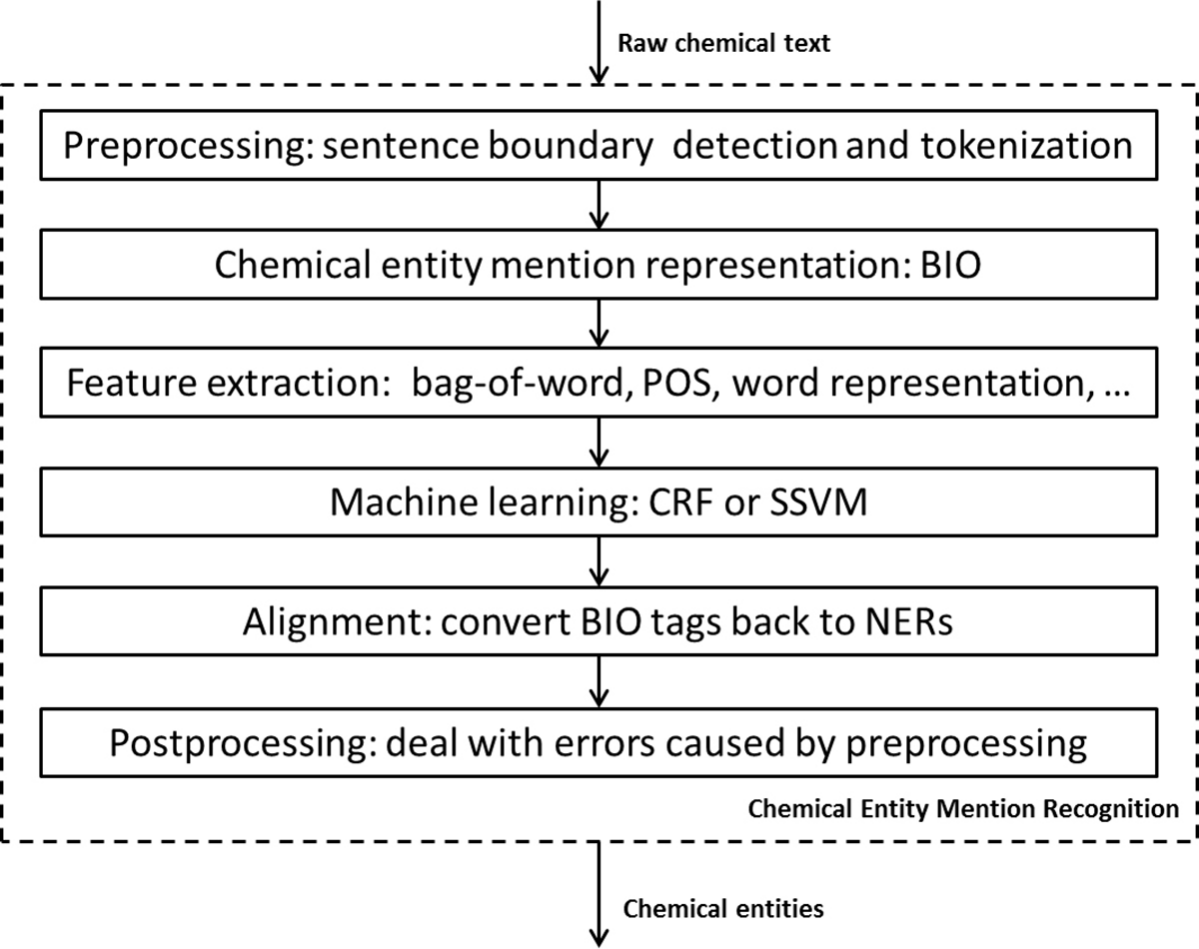
**The architecture of our system for the CEM subtask**.

Figure 1 shows the architecture of our systems for the CEM subtask of CHEMDNER which consists of six components. Since the supplied raw chemical text was not well formatted, we developed rule-based modules to detect the boundary of sentences and tokenize them for each abstract at first module. We then realigned the preprocessed abstract back to the original one prior to the post-processing step of the pipeline. The other components of the systems are presented in the following sections in detail.

### Dataset

The organizers collected 27,000 abstracts from the ISI Web of Knowledge relevant to various chemistry-related disciplines. 10,000 out of 27,000 abstracts were manually annotated with eight types of chemical entities based on a pre-defined guideline. The annotated abstracts were divided into three parts: a training set of 3,500 abstracts, a development set of 3,500 abstracts, and a test set of 3,000 abstracts. We used the training and development sets for system development, and evaluated our systems on the test set. The remaining 17,000 abstracts formed a test background set to avoid any manual correction of the predictions. Table 1 lists the counts of each type of entity in the training, development and test datasets.

**Table 1:** Statistics of the dataset.

Types	Training set	Development set	Test set	Entire corpus
ABBREVIATION	4,538	4,521	4,059	13,118

FAMILY	4,090	4,223	3,622	11,935

FORMULA	4,448	4,137	3,443	12,028

IDENTIFIER	672	639	513	1,824

MULTIPLE	202	188	199	589

SYSTEMATIC	6,656	6,816	5,666	19,138

TRIVIAL	8,832	8,970	7,808	25,610

NO CLASS	40	32	41	113

ALL	29,478	29,526	25,351	84,355

### Chemical entity recognition

**Figure 2:**
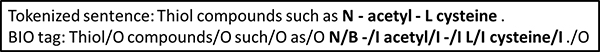
**An example of the BIO representation**.

In machine learning-based NER systems, the NER problem is usually converted into a classification problem by representing entities using specific tags. There are various representations for named entities [27] which are also suitable for chemical entities. In our study, we used BIO tags, a typical representation for named entities, to represent chemical entities, where "B", "I" and "O" denote the beginning, inside and outside of an entity respectively. Therefore, the chemical entity recognition problem is converted into a classification problem wherein the task is to assign one of the three labels to each word. Figure 2 shows an example of the BIO representation, where the chemical entity "N-acetyl-L cysteine" is represented as "N/B -/I acetyl/I -/I L/I cysteine/I" after tokenization.

The features used in our systems include bag-of-word, orthographical information, morphological information, part-of-speech (POS), document structure information, domain knowledge and WR features. They are presented in detail as below:

- Bag-of-word: unigrams, bigrams and trigrams of tokens in window of [-2, 2].

- Orthographical information: word formation information, such as capital letters, numeric characters, and their combinations. All orthographic features used in our system are shown in Table 2.

**Table 2:** Orthographic features used in our system.

Feature	Regular Expression	Feature	Regular Expression
ALLCAPS	^[A-Z]+$	MANY_NUM	^[0-9]{1,2}(,[0-9]{1,2})+$

INITCAP	^[A-Z].*	REAL_NUM	^-?[0-9]+[\.][0-9]+$

HASCAP	^.*[A-Z].*$	INDASH	^([\w+][\-]+)+\w+$

SINGLECAP	^[A-Z]$	HASDIGIT	.*[0-9].*

PUNCTATION	^[,;:\'\"]$	IS_DASH	^[-]+$

INITDIGIT	^[0-9].*	ROMAN	^[IVXDLCM]+$

SINGLEDIGIT	^[0-9]$	END_PUNC	^[.?!]$

ALPHANUM	.*[A-Za-z].*[0-9].* |.*[0-9].*[A-Za-z].*	CAPSMIX	.*[A-Z].*[a-z].* |.*[a-z].*[A-Z].*

- Morphological information: prefixes/suffixes of lengths from 2 to 5 and word shapes of tokens.

- POS: unigrams, bigrams and trigrams of POS (POS) in window of [-2, 2]. Stanford tagger was used for POS tagging http://www-nlp.stanford.edu/software/tagger.shtml.

- Document structure information: is current token in a title or not?

- Domain knowledge: whether current token includes prefixes/suffixes of chemical compounds and drugs as shown in Table 3 chemical element list and drugs found in UMLS [28], cTAKES [29] and MetaMap [30].

**Table 3:** Prefixes/suffixes of chemical compounds and drugs.

prefixes	alk, meth, eth, prop, but, pent, hex, hept, oct, non, dec, undec, dodec, eifcos, di, tri, tetra, penta, hexa, hepta
suffixes	ane, ene, yne, yl, ol, al, oic, one, ate, amine, amide

- WR features: word representation features generated by Brown clustering [24], random indexing [25] and skip-gram [26].We followed the same method as in [31] to generate unsupervised word representation features using these three methods. For detailed information, please refer to [31].

We investigated two machine learning algorithms for chemical entity recognition: CRF and Structural Support Vector Machines (SSVM). CRF is a representative sequence labeling algorithm, which is a discriminative undirected probabilistic graphical model and is suitable for NER tasks. SSVM is a large margin-based discriminative algorithm for structural data, such as sequences, bipartite graph and trees. The SSVM combines the advantages of both CRF and SVM and is also suitable for sequence labeling problems such as NER tasks.

Furthermore, we defined some simple rules to fix a number of obvious errors as indicated below:

1) If an entity starts with the end of another one, combine them together to form a new entity.

2) If an entity only contains numbers and punctuations, remove it.

3) If there is unmatched ')' in the middle of an entity, combine it with the context from the previous '(' to the start of it to form a new entity.

4) If there is unmatched '(' in the middle of an entity, combine it with the context from the end of it to the next ')' to form a new entity.

### Experiments and evaluation

In this study, we started with a baseline system that adopted features of bag-of-word, orthographic information, morphological information, POS, document structure information and domain knowledge mentioned in the previous section. Then we evaluated the effects of three types of WR features: Brown clustering-based, random indexing-based and skip-gram-based word representations, by adding each of them individually to the baseline systems. Finally, we evaluated the performance of our systems when all three types of WR features were added. All WR features were derived from the entire unlabeled abstracts (27,000) of the 2013 CHEMDNER challenge.

We used CRFsuite http://www.chokkan.org/software/crfsuite/ and SVM^hmm ^http://www.cs.cornell.edu/people/tj/svm_light/svm_hmm.html as implementations of CRF and SSVM respectively. Both of them were trained on both the training and development sets, and their parameters were optimized on the development set when the models were trained on the training set.

The basic metrics used to evaluate system performance were micro precision, recall and F-measure as shown below:

(1) $$precision=\frac{true\;positives}{true\;positives+false\;positives}$$

(2) $$recall=\frac{true\;positive}{true\;positive+false\;negetives}$$

(3) $$F-measure=\frac{2\times precision\times recall}{precision+recall}$$

where true positives corresponded to chemical entities correctly recognized, false positives corresponded to chemical entities wrongly recognized, and false negatives corresponded to chemical entities not recognized. All of them were calculated using the official evaluation tool provided by the organizers of the CHEMDNER challenge.

## Results

Table 4 shows the performance of CRF-based and SSVM-based CEM classifiers on the test sets for subtask 1 of CHEMDNER, with the three WR features added either individually or together. The SSVM-based CEM system outperformed the CRF-based CEM system when using the same features. The difference F-measure between them ranged from 0.1% to 0.4%. Addition of any type of the WR feature improved the performance of both the CRF-based and SSVM-based CEM systems. For example, when the skip-gram-based WR features were added to the baseline, the F-measure of the CRF-based CEM system was improved by about 0.5% (84.54% vs 85.05%), while the F-measure of the SSVM-based CEM system increased by about 0.2% (84.96% vs 85.20%). Among the three types of WR features, the skip-gram-based WR feature contributed to the maximal improvement as compared to the others when added to the baseline. When all the three types of WR features were added to the baseline, the F-measures of both the CRF-based and SSVM-based CEM systems were further improved, albeit only marginally. The highest F-measures achieved by the CRF-based and SSVM-based systems were 85.05% and 85.20% respectively.

**Table 4:** The performance of CRF-based and SSVM-based CEM systems when different types of WR features were used.

Feature	CRF			SSVM		
	
	Precision	Recall	F-measure	Precision	Recall	F-measure
Baseline	89.91	79.77	84.54	88.95	81.32	84.96

Baseline + BC	90.01	80.50	84.99	89.04	81.51	85.11

Baseline + RI	89.52	80.60	84.83	88.43	82.03	85.12

Baseline + SKIP	89.86	80.71	85.04	88.88	81.73	85.15

All	89.42	81.08	85.05	88.34	82.27	85.20

* BC, RI and SKIP denote three different types of word representation features: Brown clustering-based, random indexing-based and skip-gram-based word representations respectively.

## Discussion

It is not unexpected that the SSVM-based CEM system outperformed the CRF-based CEM system in the current NER task. The same result has been obtained in several

studies on other NER tasks and shown to be due to the higher recall of the SSVM-based systems when same features are used [32-34]. Use of any of the WR features was beneficial to the chemical entity recognition task. When all the three types of WR features were added, both the CRF-based and SSVM-based systems showed better performance than the systems using only one type of WR features. The effects of the WR features to CEM are similar to that reported for biomedical named entity presented in an earlier study [31]. The improvement gains from WR features are mainly due to higher recalls, indicating that WR features can improve the generalization ability of machine learning-based CEM systems.

The machine learning methods used in our systems were based on words (or tokens), which were generated by the preprocessing module shown in Figure 1. To assess the performance of machine learning methods more accurately, we studied the upper boundary performance of the machine learning-based system on the training set after preprocessing. We assigned BIO tags to all words in the training set and converted the words with tags back to chemical entities. We then compared these entities with the gold standard entities using the evaluation program and obtained the following micro precision, recall and F-measure: 97.79%, 97.84% and 97.82%, respectively. It appears that the preprocessing module affects our system to a small extent (about 2.2%). However, a quick perusal of the difference in F-measure between our system and the best system of the CHEMDNER challenge (87.39% vs 85.20%) [20] showed that it is comparable with the effect of preprocessing module. This observation has led us to determine that improvement of the preprocessing module would be a part of our future area of work or we could change our systems from word-based into char-based to avoid the performance loss caused by tokenization. Further, we also investigated the effect of the post processing module to our system. It improved the F-measure by about 0.2%.

Compared with NER tasks in the newswire domain (F-measure about 90%) [2], our reported performance on chemical entities was lower, indicating that CEM is more challenging and requires additional investigation and improvement. The possible directions of future pursuits include developing system ensemble approaches and utilizing specific patterns in the chemical domain for features.

## Conclusions

In this study, we proposed a machine learning-based system for the CEM subtask of the CHEMDNER challenge, where CRF and SSVM were used as machine learning classifiers. We also investigated the effects of three types of WR features, generated by Brown clustering, random indexing and skip-gram, on both the CRF-based and SSVM-based systems. Our experiments on the CHEMDNER challenge corpus show that the SSVM-based systems outperformed the CRF-based systems when using the same features. Addition of any of the WR features was beneficial to the CEM task. Both the CRF-based and SSVM-based systems using all the three types of WR features showed better performance than the systems only using one type of the WR feature.

## Competing interests

The authors declare that they have no competing interests.

## Authors' contributions

The work presented here was carried out in collaboration between all authors. BT, YF, XW and HX designed the methods and experiments. BT, YF, YW, MJ and JW carried out the experiments. BT, YF, XW and HX analyzed the data, interpreted the results, and wrote the paper. All authors have attributed to, seen, and approved the manuscript.

## Declarations

The publication fee of this paper is paid from JCYJ2012061315940045.

This article has been published as part of *Journal of Cheminformatics *Volume 7 Supplement 1, 2015: Text mining for chemistry and the CHEMNDER track. The full contents of the supplement are available online at http://www.jcheminf.com/supplements/7/S1.
